# Peritoneal Carcinomatosis: Intraoperative Parameters in Open (Coliseum) versus Closed Abdomen Hipec

**DOI:** 10.1155/2015/610597

**Published:** 2015-02-15

**Authors:** E. Halkia, A. Tsochrinis, D. T. Vassiliadou, A. Pavlakou, A. Vaxevanidou, A. Datsis, E. Efstathiou, J. Spiliotis

**Affiliations:** ^1^1st Department of Surgical Oncology, Metaxa Cancer Hospital, 18537 Piraeus, Greece; ^2^Peritoneal Surface Malignancy Unit, IASO General Hospital, 15562 Athens, Greece; ^3^Department of Anesthesiology, Metaxa Cancer Hospital, 18537 Piraeus, Greece; ^4^Department of Anesthesiology, Gennimatas General Hospital, 54635 Thessaloniki, Greece; ^5^Department of Surgery, General Hospital of Messolonghi, 30200 Messolonghi, Greece

## Abstract

*Background.* Peritoneal carcinomatosis (PC) is associated with a poor prognosis. Cytoreductive surgery (CRS) and HIPEC play an important role in well-selected patients with PC. The aim of the study is to present the differences in the intraoperative parameters in patients who received HIPEC in two different manners, open versus closed abdomen. *Patients and Methods.* The population includes 105 patients with peritoneal carcinomatosis from colorectal, gastric, and ovarian cancer, sarcoma, mesothelioma, and pseudomyxoma peritonei. Group A (*n* = 60) received HIPEC using the open technique and Group B (*n* = 45) received HIPEC with the closed technique. The main end points were morbidity, mortality, and overall hospital stay. *Results.* There were two postoperative deaths (3.3%) in the open group versus no deaths in the closed group. Twenty-two patients in the open group (55%) had grade III-IV complications versus 18 patients in the closed group (40%). There are more stable intraoperative conditions in the closed abdomen HIPEC in CVP, pulse rate, and systolic pressure parameters. *Conclusions.* Both methods are equal in the HIPEC procedures. Perhaps the closed method is the method of choice for frail patients due to more stable hemodynamic parameters.

## 1. Introduction

The presence of peritoneal metastases is often considered a terminal condition, not amenable to standard therapeutic management. However, cytoreductive surgery (CRS) followed by hyperthermic intraperitoneal chemotherapy (HIPEC) offers a promising alternative when implemented on well-selected patients.

HIPEC involves the rinsing of the abdominal cavity with a heated chemotherapy solution. Most regimens suggest the administration of the chemoperfusate for 60 to 120 minutes, at 42°C. The chemotherapeutic agent used depends on the site of the initial neoplasia, the most commonly used being mitomycin-c, oxaliplatin, irinotecan, and cisplatin.

**Figure 1:**
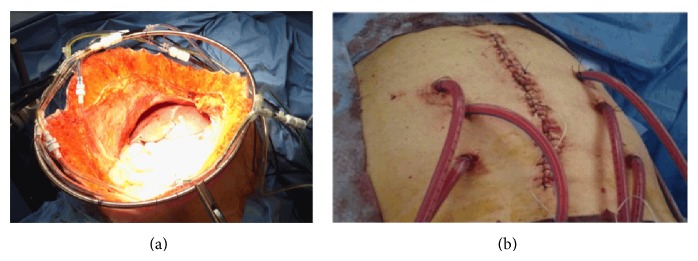
(a) The open (coliseum) technique. (b) The closed abdomen technique.

In the open technique (Figure 1(a)), the abdominal wall is elevated to create a funnel in which the chemoperfusate circulates through inflow and outflow lines attached to a pump and heating unit. On the other hand, in the closed technique (Figure 1(b)), the inflow and outflow lines are placed through separate incisions and afterwards the abdominal wall is closed before the delivery of HIPEC.

The open technique has the advantage of a more even distribution of the chemoperfusate in the abdominal cavity and is also preferred by surgeons because the formation of anastomoses is performed after HIPEC, jeopardizing less their integrity. However, its disadvantages include heat dissipation and the risk of personnel exposure to the chemotherapeutic agents, with possible toxic effects.

The closed technique, on the other hand, has been associated with uneven distribution of the perfusate in the peritoneal cavity but eliminates the exposure of the surgical team to the antineoplastic drugs. Moreover, as it has been observed in this study, it ensures more stable intraoperative conditions, making it a most appropriate choice for frail patients.

The aim of this study is to assess the differences in the intraoperative parameters during HIPEC administration with the open or the closed technique, as well as to identify perioperative morbidity and mortality.

## 2. Patients and Methods

Over a period of five years (2009–2013), a population of *n* = 105 patients was included retrospectively in this study. The origins of peritoneal carcinomatosis in those patients were colorectal, ovarian, and gastric cancer, mesothelioma, sarcoma, and pseudomyxoma peritonei. On 60 patients (Group A) the open technique was applied, while on the remaining 45 (Group B) we implemented the closed technique. Patients in both groups shared similar demographic, clinical, and therapeutic features (Table 1). The types of surgery performed are described in Table 2. Intraoperative parameters (abdominal temperature, core temperature, central venous pressure, pulse rate, systolic blood pressure, and urinary output) were recorded at 15-minute intervals from the beginning to the end of HIPEC administration (90 minutes). The outcome measures were perioperative morbidity and mortality as well as duration of hospital stay.

Patient characteristics and outcomes were analyzed by descriptive statistics. Categorical variables were compared using chi-square analysis or Fisher's exact test where appropriate. Normally distributed variables were compared using the *t*-test as appropriate; nonparametric tests were used when variables were not normally distributed. Survival was measured with the Kaplan-Meier method with *P* < 0.05 considered significant in all analysis. All statistical analyses were conducted using SPSS software (version 17.0) and Microsoft Office Excel.

## 3. Results

### 3.1. Morbidity and Mortality

In the immediate postoperative period, in the open HIPEC group, two deaths (3.3%) were recorded, while in the closed HIPEC group no postoperative deaths were recorded (NSS). Grade III and IV complications occurred in 22 patients from the open HIPEC group (55%) and in 18 patients from the closed HIPEC group (40%) (NSS). The complications are reported in Table 3.

### 3.2. Hospital Stay

Mean duration of hospital stay was 8.7 days in the open HIPEC group versus 9.1 days in the closed HIPEC group (NSS).

### 3.3. Intraoperative Parameters

**Figure 2:**
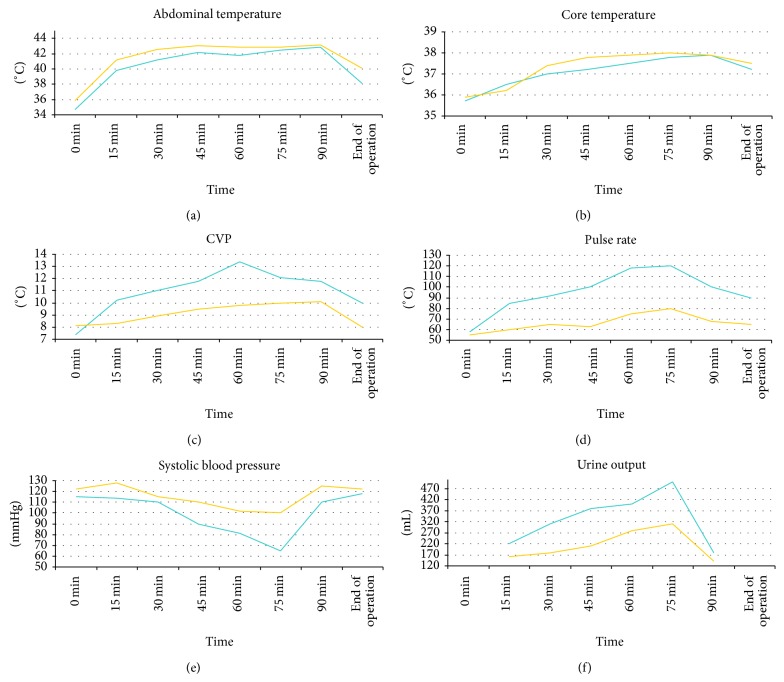
(a) Abdominal temperature. Blue = open, yellow = closed. (b) Core temperature. Blue = open, yellow = closed. (c) Central venous pressure. Blue = open, yellow = closed. (d) Heart rate. Blue = open, yellow = closed. (e) Systolic blood pressure. Blue = open, yellow = closed. (f) Urine output. Blue = open, yellow = closed.

Haemodynamic parameters recorded during the administration of HIPEC are presented in Table 4. No statistically significant differences were observed in any of the parameters studied, that is, abdominal temperature, core temperature, central venous pressure, heart rate, systolic blood pressure, and urinary output (Figures 2(a)–2(f)).

## 4. Discussion

Delivery of hyperthermic intraperitoneal chemotherapy can be safely performed using either the open or the closed technique, without significant difference in operative time or efficacy [1].

This study has shown that there are no significant differences in the postoperative morbidity and mortality with the implementation of either technique. While the analysis of the haemodynamic parameters evaluated did not yield any statistical significance either, it appears that the closed technique is associated with more stable intraoperative conditions, exposing the patient to a lesser stress. This proves to be helpful especially in frail patients, with suboptimal preoperative status (older age, comorbidities, and cachexia), suggesting the application of the closed technique in those patients.

### 4.1. Haemodynamic Parameters and Morbidity

Regarding the haemodynamic monitoring, the present study has shown that parameters such as the abdominal and core temperatures, central venous pressure, heart rate, systolic blood pressure, and urine output do not differ significantly with the two techniques.

These findings are in accordance with those of Pascual-Ramírez et al. who did not detect any difference in haemodynamic parameters during CRS and HIPEC when describing the closed technique in ovarian cancer patients, as it was also known from the study of Desgranges et al. [2, 3]. Similarly, in the Pascual-Ramírez series there was not a rise in body temperature or a disturbance in renal function, as in our study and the one conducted by Schmidt et al. [3, 4].

In our series, no difference was observed in heart rate between the open and the closed techniques. In the Pascual-Ramírez study, an increase in heart rate was reported, attributed to increased vasodilatation and relative volume deficit due to heat increase [3]. However, this finding is not present in our study, possibly due to the decreased fluid turnover that occurs in the closed method as opposed to the open one and the positive fluid balance perioperatively. Indeed, we observed more stable perioperative conditions with the closed technique, not statistically significant to those of the open one, however with narrowest ranges. This observation cannot be evaluated with statistic methods, perhaps owing to a small statistic sample being a limitation of our study.

A retrospective analysis of 78 patients undergoing cytoreductive surgery and HIPEC demonstrated a large intraoperative fluid turnover, increased airway pressure, and central venous pressure (due to the increased intra-abdominal pressure with the closed technique), while increased body temperature resulted in a mild metabolic acidosis [4].

According to the findings of another prospective study of 60 patients, haemodynamic disturbances occurred during HIPEC administration, characterized by an increase in heart rate and cardiac output and a decreased systemic vascular resistance on account of increased body temperature and decreased effective circulating volume. Urinary output showed a decreasing tendency over time [5].

A recent study by Facy et al., conducted on a swine model, reported that increased intra-abdominal pressure when applying the closed HIPEC technique resulted in tachycardia, a decrease in blood pressure despite more aggressive fluid resuscitation, and an increase in ventilation pressure [6].

Intraoperative parameters may be associated with postoperative outcome, in terms of morbidity and mortality, as demonstrated by a previous study conducted by our team [7]. However, these parameters do not correspond with long-term survival outcomes.

Postoperative ICU admission is often considered protocol after cytoreductive surgery and HIPEC, mainly due to the need for haemodynamic surveillance and stabilization after this major operation. However, it was recently reported that there was no difference in the rate and degree of complications observed in patients who were admitted to the ICU, noting that ICU admission should not be standardized but should be based on individual patient characteristics [8].

Most frequent complications of the open technique, as identified by a previous study by our team, were pulmonary complications, gastrointestinal fistulae, haematologic toxicity, and postoperative haemorrhage [9].

### 4.2. Efficacy

A comparative study between the two techniques by Ortega-Deballon et al., conducted on an animal model, identified that good thermal homogeneity was reached with both techniques; however better chemotherapeutic absorption and tissue uptake were achieved with the open technique [10].

Even with the application of high pressure with the closed technique, hypothesized to increase drug tissue penetration, the open technique still seems to attain better intraperitoneal distribution and enhanced tissue uptake [6].

The same team had previously reported a combined technique of closed HIPEC with open abdomen, utilizing a latex wall expander and a hand-access port (similar to those used in laparoscopic surgery), in an attempt to minimize personnel exposure to the chemoperfusate [11].

### 4.3. Personnel Safety

The issue of personnel safety arises with the administration of HIPEC, especially when implementing the open technique, as exposure to chemotherapeutics may result in toxic and possibly mutagenic effects on the surgical team.

Recently, it has been reported that no platinum was detected in the internal aspect of surgical gloves with neither the closed nor the open method of intraperitoneal delivery of oxaliplatin [6].

A previous study evaluated the risk of exposure to platinum in members of the surgical team and demonstrated minimal concentration in the blood and urine of the personnel, below safety threshold [12].

This suggests that, with either method, HIPEC is safe for the operating theatre personnel, given that standard protective measures are taken.

### 4.4. Laparoscopic HIPEC

A most novel method of administrating intraperitoneal chemotherapy is the laparoscopic approach [13]. In terms of efficacy, an animal study demonstrated increased drug perfusion with the laparoscopic technique [14]. Two recent cohorts of patients treated with laparoscopic cytoreductive surgery and HIPEC versus laparotomy presented no significant differences in postoperative morbidity and mortality between the two approaches, identifying laparoscopic HIPEC as a safe and efficient alternative [15, 16].

The consensus statement issued by the Peritoneal Surface Oncology Group International after the meeting in Milan in 2006 reached the conclusion that the best technique to deliver HIPEC is the open one, without sufficient evidence in the literature to prove the superiority of one technique over the other regarding outcome, morbidity, and personnel safety [17, 18].

## 5. Conclusion

Both the open and the closed abdomen technique are safe and efficient methods of HIPEC delivery in the treatment of peritoneal carcinomatosis, causing no significant haemodynamic disturbances, and are of equal morbidity and mortality. However, the more stable conditions ensured by the closed technique make it more appropriate for frail patients.

## Figures and Tables

**Table 1:** Patient characteristics, *n* = 105.

	Open HIPEC group (Group A)		Closed HIPEC group (Group B)	
*n*	60		45	
Mean age	58.3		58.1	

	*n*	%	*n*	%

Origin of peritoneal carcinomatosis				
Colorectal cancer	17	28.3	13	28.9
Ovarian cancer	14	23.3	9	20
Gastric cancer	6	10	4	8.8
Mesothelioma	8	13.3	8	17.8
Sarcoma	3	5	5	11.1
Pseudomyxoma	12	20	6	13.3
Ascites				
Yes	18	30	16	35.6
No	42	70	29	64.4
Peritoneal carcinomatosis index				
PCI < 5	7	11.7	8	17.8
5 ≤ PCI < 10	24	40	12	26.7
PCI ≥ 10	29	48.3	25	55.6
Completeness of cytoreduction				
CC-0	39	65	32	71.1
CC-1	12	20	7	15.6
CC-2	9	15	6	13.3

**Table 2:** Operations performed.

	Open		Closed	
	*n*	%	*n*	%
Splenectomy	8	13.3	7	15.6
Cholecystectomy	60	100	45	100
Omentectomy	60	100	45	100
Hysterectomy	26	43.3	11	24.4
Gastrectomy	9	15	7	15.6
Complete colectomy	19	31.7	8	17.8
Douglas resection	29	48.3	14	31.1
Small bowel resection	43	71.7	32	71.1
Partial colectomy	15	25	8	17.8
Total	**60**	**100**	**45**	**100**

**Table 3:** Complications.

			Open		Closed		
			*n*	%	*n*	%	
Grade I	No intervention required for resolution	Nausea, vomiting, metabolic acidosis, neutropenia, and ileus	9	15	10	22.2	NSS

Grade II	Medical treatment sufficient for resolution	Reintubation, blood product transfusion, and pneumonia	29	48.3	17	37.8	NSS

Grade III	An invasive intervention, for example, radiological intervention, required for resolution	Intra-abdominal collection	18	30	16	35.5	NSS

Grade IV	Urgent definitive intervention, for example, returning to the OR or to surgical ICU, required for resolution	Reoperation, readmission to ICU	4	6.7	2	4.4	NSS

Total			**60**	**100**	**45**	**100**	

**Table 4:** Haemodynamic parameters during HIPEC.

Time		0 min	15 min	30 min	45 min	60 min	75 min	90 min	End of operation
Abdominal temperature	Open	34.7 ± 1.6	39.8 ± 1.9	41.2 ± 2.4	42.2 ± 2.1	41.8 ± 2.1	42.4 ± 2.4	42.8 ± 2.1	38.1 ± 1.8
	Closed	35.9 ± 1.8	41.2 ± 1.9	42.5 ± 2.1	43.0 ± 2.3	42.8 ± 2.0	42.8 ± 5.0	43.1 ± 2.0	40.1 ± 2.0

Core temperature	Open	35.7 ± 1.2	36.5 ± 1.0	37.0 ± 1.6	37.2 ± 1.4	37.5 ± 1.6	37.8 ± 1.8	37.9 ± 1.6	37.2 ± 1.8
	Closed	35.9 ± 1.1	36.2 ± 1.2	37.4 ± 1.4	37.8 ± 2.0	37.9 ± 1.4	38.0 ± 2.0	37.9 ± 1.4	37.5 ± 1.6

CVP	Open	7.4 ± 2.1	10.2 ± 2.3	11 ± 2.8	11.8 ± 3	13.4 ± 2.9	12.1 ± 3.0	11.8 ± 2.0	10 ± 1.5
	Closed	8.1 ± 1.7	8.3 ± 1.7	8.9 ± 2.1	9.5 ± 2.4	9.8 ± 1.9	10 ± 1.9	10.1 ± 1.9	8 ± 1.1

Pulse rate	Open	58 ± 10	85 ± 11	92 ± 12	100 ± 15	118 ± 12	120 ± 11	100 ± 13	90 ± 9
	Closed	55 ± 10	60 ± 14	65 ± 12	63 ± 18	75 ± 16	80 ± 10	68 ± 18	65 ± 13

Systolic blood pressure	Open	115 ± 17	114 ± 12	110 ± 10	90 ± 10	81 ± 18	65 ± 20	110 ± 12	118 ± 20
	Closed	122 ± 10	128 ± 8	115 ± 17	110 ± 14	102 ± 20	100 ± 15	125 ± 10	122 ± 18

Urinary output (mL)	Open		220	310	380	400	500	180	
	Closed		160	180	210	280	310	140	

## References

[1] González-Moreno S., González-Bayón L. A., Ortega-Pérez G. (2010). Hyperthermic intraperitoneal chemotherapy: rationale and technique. *World Journal of Gastrointestinal Oncology*.

[2] Desgranges F.-P., Steghens A., Rosay H. (2012). Epidural analgesia for surgical treatment of peritoneal carcinomatosis: a risky technique?. *Annales Francaises d'Anesthesie et de Reanimation*.

[3] Pascual-Ramírez J., Sánchez García S., González Ruiz de la Herrán F. (2014). Security and efficiency of a closed-system, turbulent-flow circuit for hyperthermic intraperitoneal chemotherapy after cytoreductive ovarian surgery: perioperative outputs. *Archives of Gynecology and Obstetrics*.

[4] Schmidt C., Creutzenberg M., Piso P., Hobbhahn J., Bucher M. (2008). Peri-operative anaesthetic management of cytoreductive surgery with hyperthermic intraperitoneal chemotherapy. *Anaesthesia*.

[5] Rankovic V. I., Masirevic V. P., Pavlov M. J. (2007). Hemodynamic and cardiovascular problems during modified hyperthermic intraperitoneal perioperative chemotherapy. *Hepato-Gastroenterology*.

[6] Facy O., Combier C., Poussier M. (2015). High pressure does not counterbalance the advantages of open techniques over closed techniques during heated intraperitoneal chemotherapy with oxaliplatin. *Surgery*.

[7] Spiliotis J., Vaxevanidou A., Datsis A., Rogdakis A., Kekelos S. (2010). Peritoneal carcinomatosis: intra-operative and post-operative assessment of patients undergoing cytoreduction and HIPEC. *Hepato-Gastroenterology*.

[8] López-Basave H. N., Morales-Vasquez F., Mendez-Herrera C. (2014). Intensive care unit admission after cytoreductive surgery and hyperthermic intraperitoneal chemotherapy. Is it necessary?. *Journal of Oncology*.

[9] Spiliotis J. D., Rogdakis A., Vaxevanidou A., Datsis A., Zacharis G., Christopoulou A. (2009). Morbidity and mortality of cytoreductive surgery and hyperthermic intraperitoneal chemotherapy in the management of peritoneal carcinomatosis. *Journal of B.U.ON.*.

[10] Ortega-Deballon P., Facy O., Jambet S. (2010). Which method to deliver hyperthermic intraperitoneal chemotherapy with oxaliplatin? An experimental comparison of open and closed techniques. *Annals of Surgical Oncology*.

[11] Benoit L., Cheynel N., Ortega-Deballon P., Giacomo G. D., Chauffert B., Rat P. (2008). Closed hyperthermic intraperitoneal chemotherapy with open abdomen: a novel technique to reduce exposure of the surgical team to chemotherapy drugs. *Annals of Surgical Oncology*.

[12] Mahteme H., Andréasson S. N., Anundi H., Thorén S.-B., Ehrsson H. (2010). Is platinum present in blood and urine from treatment givers during hyperthermic intraperitoneal chemotherapy?. *Journal of Oncology*.

[13] Halkia E. A., Kyriazanos J., Efstathiou E., Spiliotis J. D. (2011). Laparoscopic hyperthermic intraperitoneal chemotherapy for the management of advanced peritoneal carcinomatosis. *Hepato-Gastroenterology*.

[14] Gesson-Paute A., Ferron G., Thomas F., de Lara E. C., Chatelut E., Querleu D. (2008). Pharmacokinetics of oxaliplatin during open versus laparoscopically assisted heated intraoperative intraperitoneal chemotherapy (HIPEC): an experimental study. *Annals of Surgical Oncology*.

[15] Fish R., Selvasekar C., Crichton P. (2014). Risk-reducing laparoscopic cytoreductive surgery and hyperthermic intraperitoneal chemotherapy for low-grade appendiceal mucinous neoplasm: early outcomes and technique. *Surgical Endoscopy and Other Interventional Techniques*.

[16] Passot G., Bakrin N., Isaac S. (2014). Postoperative outcomes of laparoscopic vs open cytoreductive surgery plus hyperthermic intraperitoneal chemotherapy for treatment of peritoneal surface malignancies. *European Journal of Surgical Oncology*.

[17] Esquivel J. (2009). Technology of hyperthermic intraperitoneal chemotherapy in the United States, Europe, China, Japan, and Korea. *Cancer Journal (Sudbury, Mass)*.

[18] Glehen O., Cotte E., Kusamura S. (2008). Hyperthermic intraperitoneal chemotherapy: nomenclature and modalities of perfusion. *Journal of Surgical Oncology*.

